# Self-guided virtual reality therapy for social anxiety disorder: a study protocol for a randomized controlled trial

**DOI:** 10.1186/s13063-022-06320-x

**Published:** 2022-05-12

**Authors:** Anne Sophie Hildebrand, Kati Roesmann, Jari Planert, Alla Machulska, Esra Otto, Tim Klucken

**Affiliations:** Department of Clinical Psychology and Psychotherapy, University of Siegen, Obergraben 23, 57072 Siegen, Germany

**Keywords:** Virtual reality, Social Anxiety disorder, Behavioral experiments, Exposure, E-health, Application-based therapy

## Abstract

**Background:**

Social anxiety disorder (SAD) is a highly prevalent mental disorder associated with enormous stress and suffering. Cognitive behavior therapy (CBT) is the first-line treatment for SAD, yet its accessibility is often constrained with long waiting times. Digital therapeutic applications, including psychoeducation and self-guided behavioral experiments in virtual reality (VR), could facilitate access and reduce waiting times. The study aims to investigate if ultra-short-time therapy involving self-guided digital therapeutic applications with VR components can reduce the severity of SAD.

**Methods:**

Forty SAD patients will participate in this randomized controlled trial. Half will get access to a self-guided, digital therapeutic application with exposure-based behavioral experiments in VR, while the other half will receive a control treatment. Both treatments include four therapeutic appointments. Changes in the severity of SAD will be measured after each appointment and on a 6-week follow-up assessment and will be compared between groups, with the change in SAD measured at baseline- and post-assessment as primary outcome.

**Discussion:**

Self-guided digital therapeutic applications including ultra-short-time therapy combined with VR could help reduce the waiting time for patients and relieve the health system. The results of this study may inform psychotherapists regarding the potential of self-guided digital therapeutic applications including exposure-based behavioral experiments in VR for SAD and will provide important insight for future research on VR therapy.

**Trial registration:**

Current Controlled Trials ISRCTN18013983. Registered on 1 February 2022.

## Background

Social anxiety disorder (SAD) is a highly prevalent disorder. With 12-month prevalences ranging between 1.5 and 7.1.% and lifetime prevalences between 2.5 and 12.1% [1], SAD is associated with a high load on the health system, enormous economic costs because of therapy and absenteeism [2], and a high impairment for affected individuals [1].

Social anxiety is characterized by the anxiety of being in the center of attention or of behaving embarrassingly. Thereby, blushing or trembling, fear of vomiting, and the urge of micturition or defecation are common symptoms [3]. These symptoms mainly appear in anxiety-inducing situations or when thinking about those situations and are associated with avoidance behavior, e.g., avoidance of social interactions or performing in front of others [4, 5]. When situations are not avoided, individuals engage in safety behavior (e.g., avoiding eye contact, wearing a scarf to hide symptoms of blushing, etc.) [6]. Safety behavior has three main problems: (1) individuals do not have correcting experiences about their expectations and worries, (2) symptoms of anxiety may increase, and (3) others might respond negatively [6]. Both experienced anxiety and avoidance cause individuals to experience significant impairment in everyday life [1].

Cognitive-behavioral therapy (CBT) is the first-line treatment for SAD [7]. Thereby, core therapeutic techniques are psychoeducation [8], exposure [9], and behavioral experiments [10]. During exposure-based behavioral experiments, patients are instructed to omit avoidance or safety behaviors and face the feared situation to test their pathogenic beliefs and to modify dysfunctional cognitive processes [7, 10].

Despite medium to large effect sizes of standard CBT for SAD [7], less than a quarter of individuals with SAD receive psychological or psychiatric treatment in high-income countries [1]. This has several reasons. First, a study by the Federal Chamber of Psychotherapists in Germany found an average waiting time of 20 weeks for psychotherapy [11]. Second, pre-treatment latencies contribute to pre-treatment attrition rates of up to 30.4% for individuals with SAD [12]. These numbers are worrisome, as untreated SAD mostly becomes chronic and spontaneous remissions seem to be the exception rather than the rule [13].

Technological advances in the field of e-health, like virtual reality and application-based health interventions, may help to overcome these problems. Application-based interventions including virtual reality exposure therapy have been shown to be effective in clinical settings [14]. For SAD, exposure-based VR therapy has been successfully applied [15–17]. VR therapy can include behavioral experiments (e.g., giving a presentation in front of a virtual audience to test out phobia-related expectations) [18]. It can reduce patients’ social anxiety and improve their quality of life [19]. Other advantages of exposure-based VR therapy are as follows: (1) relevant social situations can be simulated and are easier to implement [15], (2) simulations and their difficulty can be matched to the patient’s individual needs [20], and (3) it has higher acceptance and lower refusal rates than exposure in vivo [21]. For specific phobia and smoking, self-guided digital VR therapy has proven to be effective [14, 22, 23]. To access these benefits in SAD, application-based therapeutic interventions that may include psychoeducation and VR components to model feared situations can be used. These interventions can support ultra-short-time therapy, in which most of the therapeutic work will be provided within application. Even though application-based therapeutic interventions are a promising approach to reduce the enormous load on the health system, studies testing their efficacy are still sparse.

To extend the current knowledge and to test the efficacy of application-based short-term therapy including behavioral experiments in virtuo, one group of patients (experimental group) will get access to a digital (VR) application and four appointments with a psychotherapist, while the second group (control group) will get a control treatment composed of four appointments with a psychotherapist. We expect that patients who receive application-based treatment will show less symptoms of social anxiety at a post-assessment compared to a baseline assessment and at an interim, a post, and a 6-week follow-up assessment, compared to the control group. To test a clinically relevant change, the remission rates of patients between both groups will be compared at the follow-up assessment.

### Trial design

In the present randomized controlled trial, changes in SAD symptoms (post vs. baseline, interim vs. baseline, follow-up vs. baseline) will be compared between a group receiving the digital (VR) application and a control group in a superiority design. The study employs a 2 (condition: application-based vs. control treatment) × 4 (time: baseline assessment, interim assessment, post assessment, and 6-weeks-follow-up) design.

## Methods/design

### Participants

Patients with SAD will be recruited from the general population and routine care at the Outpatient Center for Psychotherapy of the University of Siegen, Germany. Participants will be recruited through newspaper reports, advertisements, and radio. Interested participants will be invited to take part in a telephone interview to access inclusion and exclusion criteria. Participants will be included if they fulfill the criteria for social anxiety disorder as a primary disorder. Criteria will be tested with a short diagnostic interview for mental disorders (Mini-Dips [24]). Participants must be at least 18 years old and not receiving therapy yet. Due to exposure-based behavioral experiments in virtual reality, participants who experienced a stroke or coronary disease in the past or are diagnosed with angina pectoris, cardiac arrhythmias, hypertension, asthma, or a chronic obstructive pulmonary disease, strong visual disorders or epilepsy or seizures in the past, or (possible) pregnancy will be excluded. Also, participants with psychological disorders with organic cause, vertigo, vestibular impairments, psychological or behavioral problems caused by psychoactive substances, schizophrenia, schizotypal or delusional disorders, severe depression or mania, acute suicidal tendencies, or lack of capability to negotiate a no-suicide agreement will be excluded.

### Sample size

**Fig. 1:**
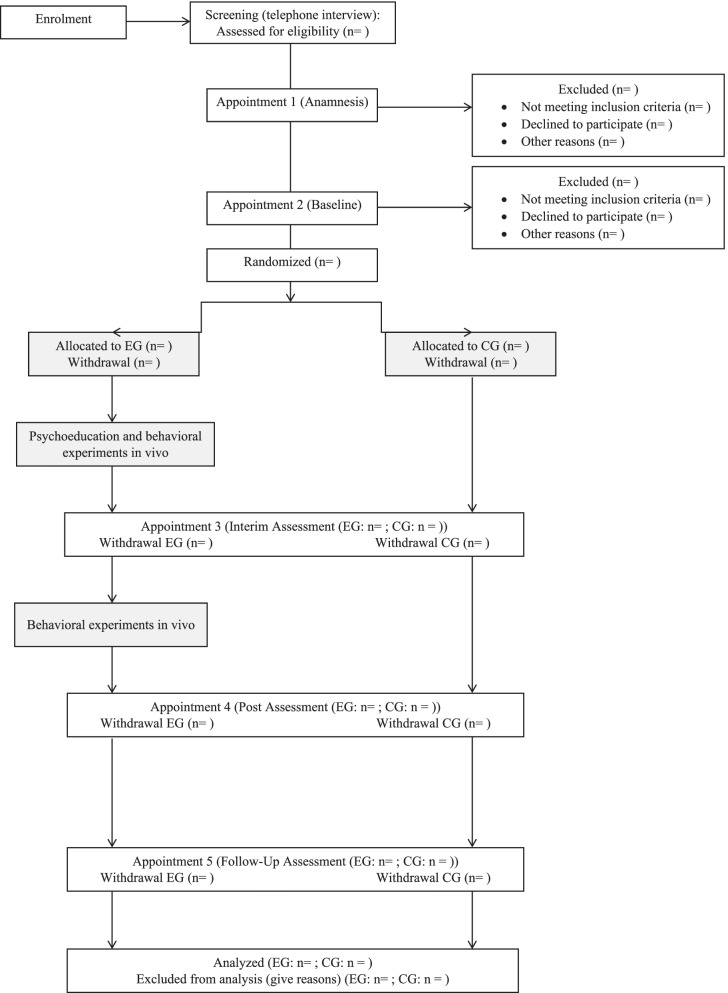
The consort flow diagram. EG, experimental group; CG, control group; white, the same for both groups; grey, different for the groups

In previous studies, medium to large effect sizes were found for exposure-based VR therapy when compared to a control treatment without behavioral exposure for self-report measures [25]. For self-guided therapy-derived interventions, small to medium effect sizes and a medium mean effect size were found for self-report measures [26]. Hence, we anticipate a medium effect size for the primary outcome of the present study. An a priori power analysis was conducted for the primary outcome (change in social anxiety symptoms as assessed by means of the Social Anxiety and Social Competence Deficits questionnaire; SASKO [27], measured at baseline- and post-assessment) using G*Power 3.1 [28] for a 2 × 4 repeated measures ANOVA (Cohen’s *d* = .50, *α* = .05, power = .80). The results indicated a required total sample size of 24 participants. Due to expected attrition, 40 participants will be included in the study, that is 20 per condition. Figure 1 shows a CONSORT flow diagram of the recruitment process.

## Methods and materials

### Procedure

#### Screening

To pre-select the participants, a screening via telephone will be used, including SAD symptoms as outlined in the Mini-DIPS and inclusion and exclusion criteria. Participants that do not fulfill the criteria will be excluded and offered to be put on the waiting list of the Outpatient Center for Psychotherapy of the University of Siegen. The screening will be conducted by research assistants, trained by a psychotherapist. If participants fulfill the inclusion criteria, they will be invited to a first appointment with a certified psychotherapist.

#### First appointment (anamnesis)

During the first appointment with their psychotherapist (100 min), participants will receive information on the study and provide written informed consent. Further, patients’ motivation for seeking therapeutic help will be explored, and core symptoms and a biographical anamnesis will be obtained.

#### Second appointment (baseline assessment)

At a second appointment (around 100 min), a structured clinical interview, the Mini-DIPS, will be conducted by a psychotherapist to verify the diagnosis of SAD, to explore comorbid psychological disorders, and to test for psychological disorders leading to exclusion from this study. If eligible for participation, patients will be assigned to the experimental group (EG) or the control group (CG) according to a computer-generated randomization scheme and participate in an online survey, conducted by trained research assistants. Afterwards, patients randomized to the EG will receive their prescription for the application (Invirto 1.17.0, Sympatient, Hamburg; class I after the Medical Device Directive 93/42/EWG [29]). The EG will be asked to start the intervention, while the CG will be asked to wait until the next appointment.

#### Self-guided phase I

Participants in the EG will be provided with the prescribed therapeutic application [29] and the VR glasses to perform the behavioral experiments in virtuo. They will be instructed to complete the first units (Part 1: Psychoeducation and behavioral experiments in virtuo) within 6 weeks after the baseline assessment. Each unit is split into psychoeducation and exercises. Psychoeducation includes information on SAD and mechanisms involved in its development and maintenance (e.g., triggers, safety behavior, and dysfunctional cognitions). The exercises include relaxation exercises, behavioral analyses, and behavioral experiments in virtuo. The virtual elements can last up to 90 min: first, participants are familiarized with the virtual psychotherapist and the virtual reality experience. Afterwards, patients are confronted with two virtual anxiety-inducing contexts (a job application and a talk). These exercises are designed to train patients to identify safety behaviors (e.g., via video feedback), to test (and falsify) anxiety-inducing worries and distorted expectations, and to gain new experiences (e.g., anxiety with and without safety behaviors) with anxiety-inducing virtual situations. The CG will not receive a specific instruction for this phase.

#### Third appointment (interim assessment)

Six weeks after baseline assessment, that is after completion of part 1 (Psychoeducation and behavioral experiments in virtuo) for the EG, patients are invited to a third therapeutic appointment with a psychotherapist. During this appointment, patients in the EG will plan and practice the upcoming behavioral experiments in real social situations. To control for unspecific treatment effects in the EG that might be caused by contact with a psychotherapist, the CG will also meet a psychotherapist.

**Fig. 2:**
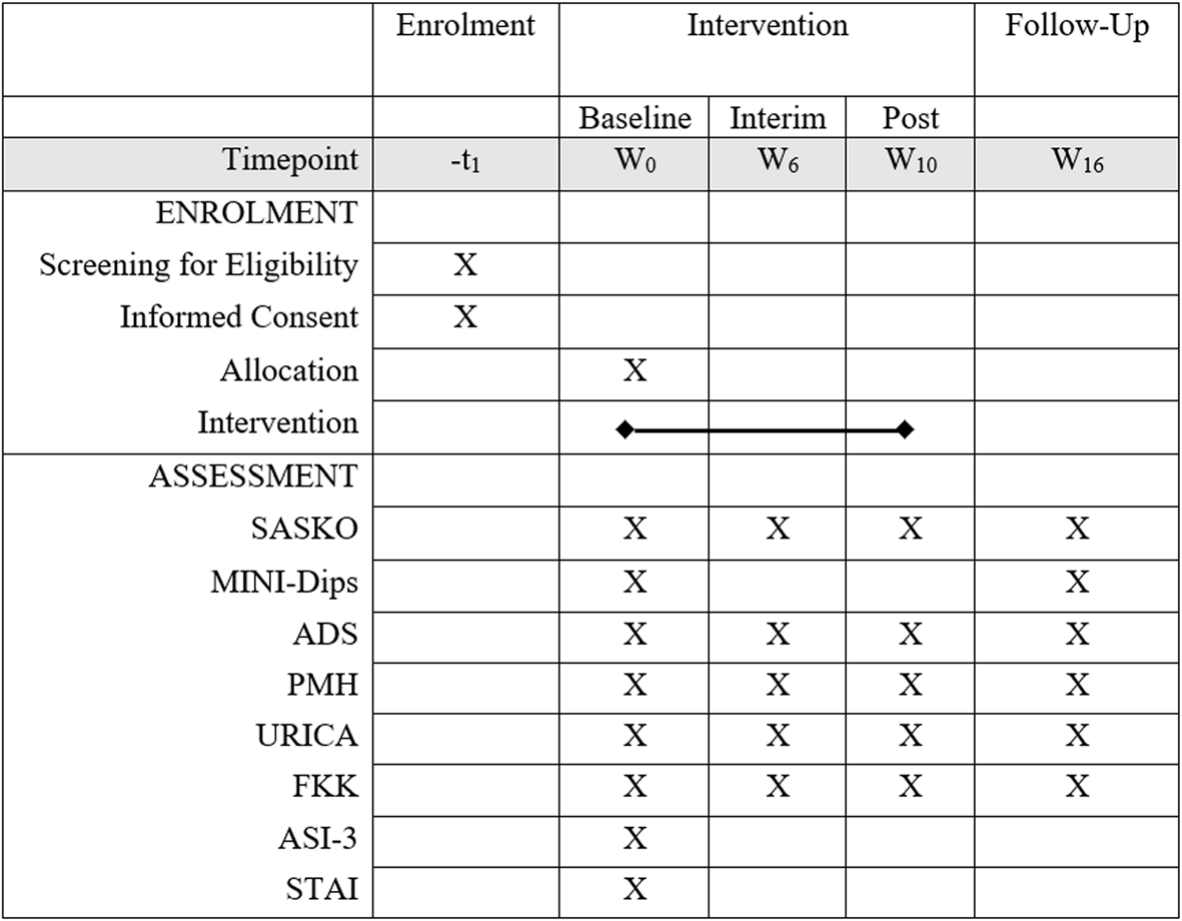
Schedule of enrollment, intervention, and assessment. Measurement schedule, including all outcome measures. An X in the corresponding box indicates that a measurement takes place at a certain time point. W, weeks after allocation; SASKO, Questionnaire for Social Anxiety and Social Competence Deficits; Mini-Dips, short diagnostic interview for mental disorders; ADS, Center for Epidemiological Studies Depression Scale; PMH, Positive Mental Health; URICA, University of Rhode Island Change Assessment Scale; FKK, Questionnaire on Beliefs in Competency and Control; ASI-3, Anxiety Sensitivity Index; STAI, State-Trait-Anxiety-Inventory

The CG will be informed on stress-reduction and relaxation techniques and the interim assessment will be filled in. The topic of stress and relaxation was chosen because it is not specific to the disorder. Afterwards, patients will fill in the questionnaires of the interim assessment (see Fig. 2).

The interim assessment serves two purposes: first, it aims at assessing the effects of the psychoeducation and VR therapy (+ psychotherapist appointment) compared to the control treatment. Second, it allows for an investigation of add-on effects of the subsequent self-guided behavioral experiments in vivo.

#### Self-guided phase II

For the next 4 weeks, participants in the EG will be instructed to complete behavioral experiments in vivo while participants in the CG will be instructed to wait.

#### Fourth appointment (post assessment)

During the fourth appointment (around 100 min, 4 weeks after the interim assessment), patients of both groups will meet their psychotherapist to reflect on their experiences. Afterwards, the questionnaires of the post assessment will be filled in (see Fig. 2).

#### Follow-up assessment

At the follow-up appointment (6 weeks after the post assessment, around 100 min), both EG and CG will fill in the questionnaires, and a Mini-DIPS will be conducted by the psychotherapist to assess remission status. Afterwards, the CG will get the prescription for the application.

#### Participant retention

All participants can get feedback on their process from their therapists after the follow-up, and they will be reminded of appointments by the research assistants. At each appointment, therapists and research assistants will give verbal encouragement to complete application units to benefit most from the proposed treatment. To promote participant retention in the control group, participants will be given the prescription for the therapeutic application after the follow-up.

### Outcome measures

#### Primary outcome

The primary outcome measure will be the total score of the German version of the SASKO [27] measured at baseline assessment and post assessment (post-assessment–baseline assessment). The SASKO is a self-report questionnaire that contains the symptoms of SAD. It has four subscales: (1) anxiety of speaking and being in the center of attention (12 items), (2) fear of rejection (10 items), (3) interaction deficits (10 items), and (4) information processing deficits (8 items) and a total score (40 items). All items are judged on a 4-point Likert scale ranging from 0 (“never”) to 3 (“always/most of the time”). It has a high internal consistency (Cronbach’s *α* = .92) and good validity [27]. Participants will be presented with the SASKO at four time points. These time points are the baseline assessment (around 2 weeks after enrollment), interim assessment (6 weeks after baseline), post-assessment (10 weeks after baseline), and follow-up assessment (16 weeks after baseline).

#### Secondary outcome

Remission will be used as a clinically relevant outcome. Remission will be assessed by the Mini-Dips [24], a reliable (Kappa = .94 for anxiety disorders) and valid instrument, that is often used in clinical studies [30], on follow-up assessment (16 weeks after baseline).

#### Exploratory variables

Effects of the treatment on additional clinically relevant measures (e.g., anxiety, depression, control beliefs, etc.), as well as the role of potential moderators/mediators, will be tested in exploratory analyses. Therefore, the following measures will be obtained at all four-time points: (1) the Questionnaire on Beliefs in Competency and Control (FKK) [31], (2) the German version of the Center for Epidemiological Studies Depression Scale (ADS) [32], (3) Positive Mental Health (PMH) [33], and (4) the German version of the University of Rhode Island Change Assessment Scale (URICA) [34]. At baseline, (1) the State-Trait-Anxiety-Inventory (STAI) [35] and (2) the Anxiety Sensitivity Index (ASI-3) [36] will be applied. They will only be filled in once because of the stability of the measures. The detailed procedure of measurements is listed in the schedule of enrollment, intervention, and assessment (Fig. 2).

#### Ethics statement

The study protocol (10/2021) was approved by the Ethics Committee of the University of Siegen (reference number: ER_84_2021). The study was designed in accordance with the Declaration of Helsinki, Good Clinical Practice guidelines, and the SPIRIT reporting guidelines. During the run of the study, ethical, legal, and social aspects will be anticipated and addressed. Participants will provide written informed consent to the experimental procedure prior to inclusion in the study. Participation will be entirely voluntary, and participants will have the right to withdraw their consent at any time. Data will be pseudonymized with a trial identification number. It will be saved on a secure, self-encrypting database and can only be accessed by the responsible researchers. Participants will not be financially compensated for their participation in this low-risk intervention. No major adverse events are anticipated. Yet, as a minor anticipated event, motion sickness because of virtual reality is possible. In case of any unforeseen adverse events or a deterioration of symptoms, participants will have the opportunity to talk to their therapist, who will then initiate necessary care steps.

#### Randomization and blinding

Participants will be randomly assigned to the experimental or the control group by a 1:1 allocation ratio. A computer-generated randomization scheme was implemented via R 4.1.1. Permuted block randomization will be used to ensure that both groups include 20 participants. Due to the study design, it is not possible to blind the participants or the psychotherapists after the allocation. Participants will be assigned to the interventions by the therapists. To avoid bias during data assessment and analysis, research assistants concerned with enrollment and data analysis will be blind to participants’ group allocations.

#### Data preparation and planned analyses

Multiple imputations of missing data will be performed for intention-to-treat (ITT) analyses [37]. Application-based treatment effects on the primary and secondary outcome measures will be tested using variance analytic methods. Potential associations between exploratory as well as demographic variables and the factor group, as well as their associations with outcome-variables, will be explored. If adequate, analyses on treatment effects on primary and secondary outcomes will be recalculated, thereby controlling for potential confounding effects.

## Discussion

SAD is a highly prevalent psychological disorder that is associated with enormous distress and suffering. Even though psychotherapy—especially cognitive behavioral therapy—is highly effective, its accessibility is constrained. Long waiting lists contribute to high pre-therapy attrition rates and chronic progressions. Self-guided digital therapeutic applications that include VR could help to address these problems.

The present study is designed to investigate the efficacy of an ultra-short-time therapy in combination with digital self-guided psychoeducation and exposure-based behavioral experiments in virtuo to improve symptoms and impairment of individuals with SAD in a routine-care context.

If proven effective, the use of application-based interventions could have many benefits for therapeutic practice. First, because of only a few appointments with therapists, more patients could be treated by these therapists in equivalent periods. This could reduce waiting times and the risk of pre-therapy attrition and chronic progressions of SAD. Second, patients can complete VR therapy independently of a psychotherapist. This could reduce the threshold for patients to seek therapeutic help, as well as organizational efforts and costs associated with exposure-based treatments. Importantly, many previous studies have suggested that exposure-based interventions employing VR are as effective as in vivo treatments [17]. Additionally, in specific phobia, not only therapist-guided but also self-guided exposure-based VR therapy was revealed effective [22, 23]. Given the lack of comparable research in other anxiety disorders, this study will advance our understanding on the potential of self-guided digital VR therapy for patients with SAD.

Overall, the study results can inform future research and clinical practice. As application-based therapy could provide a cost-effective and easy-to-access intervention that could be used as an add-on to traditional treatments and/or to provide more patients access to therapy and to reduce waiting lists, this research is highly relevant.

### Trial status

The trial was registered on 1 February 2022 (registration number ISRCTN18013983, protocol version number 1.0). The recruitment process began in March 2022 and will finish in January 2024. Any deviations from the protocol will be fully documented and the protocol will be updated in the clinical trial registry.

## Data Availability

The anonymized datasets used and/or analyzed during the current study are available from the corresponding author on reasonable request.

## Abbreviations

ADS: Center for Epidemiological Studies Depression Scale
ANOVA: Analysis of variance
ASI-3: Anxiety Sensitivity Index
CBT: Cognitive behavioral therapy
CG: Control group
EG: Experimental group
FKK: Questionnaire on Beliefs in Competency and Control
Mini-Dips: Short Diagnostic Interview for Mental Disorders
PMH: Positive Mental Health
SAD: Social anxiety disorder
SASKO: Questionnaire for Social Anxiety and Social Competence Deficits
STAI: State-Trait-Anxiety-Inventory
URICA: University of Rhode Island Change Assessment Scale
VR: Virtual reality
